# Diet, smoking and lung cancer: a case-control study of 1000 cases and 1500 controls in South-West England

**DOI:** 10.1054/bjoc.2000.1668

**Published:** 2001-03

**Authors:** S Darby, E Whitley, R Doll, T Key, P Silcocks

**Affiliations:** ICRF/MRC/BHF Clinical Trial Service Unit & Epidemiological Studies Unit, Radcliffe Infirmary, Harkness Building, Oxford, OX2 6HE; Department of Social Medicine, University of Bristol, Bristol, BS8 2PR; ICRF Cancer Epidemiology Unit, Radcliffe Infirmary, Gibson Building, Oxford, OX2 6HE; Trent Institute for Health Service Research, Queens Medical Centre, Nottingham, NG7 2UH

**Keywords:** diet, lung cancer, carotene, retinol

## Abstract

We have examined the relationship between diet and lung cancer in a case–control study of 982 cases of lung cancer and 1486 population controls in south-west England in which subjects were interviewed personally about their smoking habits and their consumption of foods and supplements rich in retinol or carotene. Analyses were performed for 15 dietary variables, including intake of pre-formed retinol and carotene. There were significant associations (*P*< 0.01) with lung cancer risk for 13 of the variables, eight of which remained after adjustment for smoking. When the 15 variables were considered simultaneously, independent significant associations remained for 5: pre-formed retinol (increased risk), and fish liver oil, vitamin pills, carrots and tomato sauce (decreased risk). It is unlikely that all five associations represent biological effects, or that they can all be explained by residual confounding by smoking, or by biases. We conclude that there is at least one as yet unidentified factor that is causally related to lung cancer risk and of considerable importance in terms of attributable risk in this population. © 2001 Cancer Research Campaign http://www.bjcancer.com


*British Journal of Cancer* (2001) **84**(5), 728–735
© 2001 Cancer Research Campaign
doi: 10.1054/ bjoc.2000.1668, available online at http://www.idealibrary.com on IDEAL
http://www.bjcancer.com

### Diet, smoking and lung cancer: a case–control study of 1000 cases and 1500 controls in South-West England

**S Darby[1], E Whitley[2], R Doll[1], T Key[3] and P Silcocks[4]**

[1]ICRF/MRC/BHF Clinical Trial Service Unit & Epidemiological Studies Unit, Harkness Building, Radcliffe Infirmary, Oxford OX2 6HE; [2]Department of Social Medicine, University of Bristol, Bristol BS8 2PR; [3]ICRF Cancer Epidemiology Unit, Gibson Building, Radcliffe Infirmary, Oxford OX2 6HE; [4]Trent Institute for Health Service Research, Queens Medical Centre, Nottingham NG7 2UH

**Summary** We have examined the relationship between diet and lung cancer in a case–control study of 982 cases of lung cancer and 1486 population controls in south-west England in which subjects were interviewed personally about their smoking habits and their consumption of foods and supplements rich in retinol or carotene. Analyses were performed for 15 dietary variables, including intake of pre-formed retinol and carotene. There were significant associations ($P < 0.01$) with lung cancer risk for 13 of the variables, eight of which remained after adjustment for smoking. When the 15 variables were considered simultaneously, independent significant associations remained for 5: pre-formed retinol (increased risk), and fish liver oil, vitamin pills, carrots and tomato sauce (decreased risk). It is unlikely that all five associations represent biological effects, or that they can all be explained by residual confounding by smoking, or by biases. We conclude that there is at least one as yet unidentified factor that is causally related to lung cancer risk and of considerable importance in terms of attributable risk in this population. © 2001 Cancer Research Campaign http://www.bjcancer.com

**Keywords:** diet; lung cancer; carotene; retinol

It is 25 years since Bjelke (1975) reported that the risk of lung cancer might be reduced by a high consumption of vitamin A. Subsequent work soon showed that the inverse association Bjelke had observed was with β-carotene and other provitamin A carotenoids, which can be converted into vitamin A in the body, rather than with retinol, which is pre-formed vitamin A (Peto et al, 1981). However, the idea that β-carotene was protective proved untenable as dietary supplements failed to produce any beneficial effect in randomized controlled trials (Lee et al, 1995; Cook et al, 2000), some even suggesting it might be harmful (Alpha-tocopherol Beta Carotene Cancer Prevention Study Group, 1994; Omenn et al, 1996). Observational studies have continued, however, to show an inverse association between lung cancer and the intake of green and yellow vegetables and carotenoids, which could be due to a beneficial effect of some other chemical (or group of chemicals) in vegetables with which carotenoids are associated. Alternatively the findings might be due (at least in part) to inadequate allowance for smoking, with which dietary habits are to some extent confounded.

We report here the findings of a study in which the latter possibility could be tested in unusual detail. The study was carried out in south-west England to quantify the effects on the risk of lung cancer of exposure to radon in the home (Darby et al, 1998). For this purpose it was essential to be able to allow for the effects of different smoking habits and it seemed also desirable to be able to allow for any effects of dietary retinol and carotene; detailed personal histories of all three were therefore obtained.

*Received 5 October 2000*
*Revised 29 November 2000*
*Accepted 29 November 2000*

*Correspondence to:* S Darby

### METHODS

### Study subjects

At each of the 5 centres in Devon and Cornwall where treatment of lung cancer is carried out, research assistants sought, over a 4-year period during 1988–1993, to interview all long-term residents of the area who were aged less than 75 years, were ethnically white, and who were referred with a suspected diagnosis of lung cancer. For each interviewed subject with suspected lung cancer, a population control subject was also interviewed. Controls were randomly selected either from lists of the local Family Health Services Authority or from electoral rolls, and were frequency matched for age, sex and broad area of residence to the patients with suspected lung cancer. Following the interview the final diagnosis of all those with suspected lung cancer was sought. Those who did not, in fact, have lung cancer were excluded from the analysis. The final number of subjects in the analysis was 2468, comprising 982 subjects with cancer of the trachea, bronchus or lung (International Classification of Diseases, 9th revision, code 162 (World Health Organization, 1975), but excluding carcinoids) and 1486 controls (see Table 1). 33% of subjects were female. The proportion of eligible subjects who were not eventually included in the study was 12% among cases and 21% among controls. Further details are given elsewhere (Darby et al, 1998; Peto et al, 2000). The original study included hospital as well as population controls. The hospital controls were selected from patients whose current admission was for a disease thought not to be related to smoking. However, a number of them had diseases that might either be caused by diet or cause a change in diet. The hospital control group was therefore not used for the main analyses of diet and lung cancer.

**Table 1** Numbers of subjects by sex and smoking status

| Smoking status | Subjects with lung cancer | | | | Control subjects | | | |
|---|---|---|---|---|---|---|---|---|
| | Male | | Female | | Male | | Female | |
| | Number | (%) | Number | (%) | Number | (%) | Number | (%) |
| Life-long non-smoker[a] | 3[d] | (0.4) | 23 | (7.3) | 195 | (19.7) | 255 | (51.3) |
| Current cigarette (<15 per day) | 128 | (19.2) | 71 | (22.5) | 110 | (11.1) | 46 | (9.3) |
| Current cigarette (15–24 per day) | 126 | (18.9) | 86 | (27.3) | 71 | (7.2) | 38 | (7.7) |
| Current cigarette (25+ per day) | 68 | (10.2) | 38 | (12.1) | 21 | (2.1) | 6 | (1.2) |
| Ex-smoker (<10 years)[b] | 146 | (21.9) | 68 | (21.6) | 162 | (16.4) | 36 | (7.2) |
| Ex-smoker (10+ years)[b] | 139 | (20.8) | 26 | (8.3) | 355 | (35.9) | 107 | (21.5) |
| Other[c] | 57 | (8.6) | 3 | (1.0) | 75 | (7.6) | 9 | (1.8) |
| Total number of subjects | 667 | (100.0) | 315 | (100.0) | 989 | (100.0) | 497 | (100.0) |

[a]Those who had never smoked as much as one cigarette per day for as long as a year, or smoked cigars or a pipe regularly for as long as a year, and who had smoked less in total than 500 cigarettes, 100 cigars or 20 oz of pipe tobacco. [b]Ex-smokers are those who had stopped smoking by the onset of their illness (lung cancers) or by the date of their interview (controls). [c]Current pipe or cigar smokers who did not smoke cigarettes and occasional smokers, i.e. those who were not lifelong non-smokers but had never smoked as much as one cigarette per day or cigars/pipe for as long as a year. [d]The number of males with lung cancer who were lifelong non-smokers is atypically low and should not be used to estimate relative risk. See Peto et al (2000) for further details.

© 2001 Cancer Research Campaign

### Information on diet and other factors

The questionnaire for this study was drawn up in the late 1980s. At that time it was thought that there might be a causal relation between intake of vitamin A (either retinol or β-carotene) and risk of lung cancer, with increased intake reducing the risk. Therefore a questionnaire was devised to ascertain the frequency with which foods containing these nutrients were eaten using a 7-point scale (never, a few times a year, about monthly, about weekly, a few times a week, about daily, a few times a day). Where appropriate, separate questions were asked about winter and summer consumption. To avoid bias caused by recent illness among subjects with lung cancer, questions were specifically asked about consumption levels one year before the interview. Questions were also asked about the time periods during which 'vitamin pills' or fish liver oil (a rich source of retinol), in either liquid or capsule form, had been taken regularly. A copy of the dietary questionnaire is available on request. This questionnaire was not validated directly, but it was similar in design to the questionnaire used in a previous study in a similar English population for which the correlation between estimated carotene intake and serum β-carotene in 55 non-smokers was +0.33 (Harris et al, 1991).

Using the questionnaire data together with standard protion sizes (Ministry of Agriculture, Fisheries and Food, 1993) and information on the composition of foods (Holland et al, 1991), estimates were made of the daily intake of 5 nutrients or proxies for nutrients: pre-formed retinol, carotene (as β-carotene equivalents (Holland et al, 1991)), 'all vegetables', green vegetables (as a proxy measure for lutein), and tomatoes (as a proxy measure for lycopene). Subjects were divided into quartiles for each of these 5 measures except for 'green vegetables', which over 60% of subjects reported eating a few times per week. For 'green vegetables', therefore, the subjects were divided into 4 intake groups such that the smallest group had as many subjects as possible. For the analyses of diet and lung cancer risk, an *a priori* decision was made to examine the results for estimated intake of the 5 nutrients or nutrient proxies mentioned above and for 8 individual food frequencies (carrots, butter, cheese, eggs, liver, margarine, milk and tomato sauce) plus vitamin pills and fish liver oil. For the individual food frequencies, subjects were also divided into 4 groups that were as nearly equal in size as possible. Taking 'vitamin pills' or fish liver oil were both assumed to involve consumption of 800 μg of retinol per day (Proprietary Association of Great Britain, 1996). Results are presented in this paper for all the 15 dietary variables that were examined.

During the interview, subjects were asked about their smoking habits at the onset of the illness that brought them to hospital (lung cancers) or currently (controls). Among the subjects with lung cancer, only 0.4% of males and 7.3% of females were lifelong non-smokers, compared with 19.7% of males and 51.3% of females among the controls (see Table 1). Subjects were also asked about their residential and occupational histories, exposure to radiotherapy and county of birth. Based on current or last job (husband's current or last job for married women) subjects were classified into 3 social class groupings: I & II, III manual and non-manual, IV & V (Office of Population Censuses and Surveys, 1980). In addition the average residential radon exposure during the 30-year period ending 5 years before the interview was estimated for all subjects, using direct measurements wherever possible. Further details are given elsewhere (Darby et al, 1998).

### Method of analysis

Associations between dietary measures and lung cancer risk were studied using the Stata statistical package (StataCorp, 1997). Relative risks were estimated by the maximum likelihood estimate of the odds ratio, based on unconditional logistic regression. Significance levels were based on the likelihood ratio test and are 2-sided, and confidence intervals were based on standard errors. Tests for trend are based on the grouped data, using the groups presented below with quantitative values 1, 2, 3, . . . etc. assigned to the groups. The analyses reported in Table 2 were repeated (i) using a finer adjustment for smoking (16 categories rather than 7), and (ii) including terms for residential radon, county district of current residence, work in a job incurring a potential lung cancer risk, exposure to radiotherapy, and birth in Devon or Cornwall. Neither of these changes made any material difference to the results.

### RESULTS

### Nutrients

After adjusting for age and sex there was a significant positive association between risk of lung cancer and intake of pre-formed retinol ($P < 0.001$). Subjects in the highest quartile of retinol intake had a relative risk (RR) of lung cancer of 1.63 (95% confidence interval (CI): 1.29, 2.05) compared with subjects in the lowest quartile (see Table 2). When adjustment was also made for smoking status the RR was altered only slightly, to 1.61 (95% CI: 1.24, 2.10), and the association remained significant ($P = 0.003$). For carotene there was a significant negative association with lung cancer when adjustment was made only for age and sex ($P < 0.001$) and subjects in the highest intake quartile had RR 0.56 (95% CI: 0.45, 0.71) compared to subjects in the lowest quartile. However, after adjustment also for smoking, the significance level became much less extreme ($P = 0.06$) and the RRs closer to unity, being 0.74 (95% CI 0.56, 0.96) for the highest intake quartile. For all vegetables the results were similar to those for carotene in that when adjustment was made only for age and sex there was a highly significant negative association with lung cancer ($P = 0.002$) and the RR for those in the highest intake quartile was considerably less than one (0.63, 95% CI: 0.50, 0.80). However, when adjustment was also made for smoking, the trend was no longer significant ($P = 0.75$) and the RR of those in the highest intake quartile was much closer to unity (0.90, (95% CI 0.69, 1.17)). For green vegetables there was little evidence of an association regardless of the method of adjustment. For tomatoes there was a significant negative association with lung cancer when adjustment was made only for age and sex ($P < 0.001$) and subjects in the highest intake quartile had RR 0.59 (95% CI: 0.47, 0.75) compared to subjects in the lowest quartile. When adjustment was also made for smoking, the significance level of the trend became somewhat less extreme ($P =$ 0.01) and the RR for subjects with the highest intake increased to 0.74 (95% CI 0.57, 0.96).

### Individual foods and supplements

Among the individual foods/supplements, there were negative associations for carrots, margarine, tomato sauce, vitamin pills and fish liver oil which remained statistically significant ($P < 0.001$, $P = 0.001$, $P = 0.001$, $P = 0.02$, and $P < 0.001$ respectively) after

 *© 2001 Cancer Research Campaign*

**Table 2** Distribution of cases and controls and relative risk of lung cancer by nutrient, food, or supplement intake, based on usual habits one year before interview. Each nutrient, food or supplement is considered separately

| Nutrient, food or supplement | Intake | No. of cases/ No. of controls | Relative risk adjusted for age and sex[a] | Relative risk adjusted for age, sex and smoking[b] |
|---|---|---|---|---|
| Pre-formed retinol (μg day$^{-1}$) | <430 | 221/396 | 1.00[c] | 1.00[c] |
| | 430– | 243/374 | 1.17 (0.93, 1.48) | 1.28 (0.98, 1.67)[d] |
| | 755– | 225/392 | 1.03 (0.82, 1.31) | 1.08 (0.83, 1.41) |
| | 1536+ | 293/324 | 1.63 (1.29, 2.05) | 1.61 (1.24, 2.10) |
| *P* value for trend | | | <0.001 | 0.003 |
| Carotene (μg day$^{-1}$) | <1305 | 316/301 | 1.00 | 1.00 |
| | 1305– | 209/408 | 0.49 (0.39, 0.62) | 0.60 (0.46, 0.78) |
| | 1904– | 230/387 | 0.57 (0.45, 0.71) | 0.69 (0.53, 0.90) |
| | 2512+ | 227/390 | 0.56 (0.45, 0.71) | 0.74 (0.56, 0.96) |
| *P* value for trend | | | <0.001 | 0.06 |
| All vegetables[e] (g day$^{-1}$) | <85 | 288/329 | 1.00 | 1.00 |
| | 85– | 222/399 | 0.63 (0.50, 0.80) | 0.77 (0.59, 0.99) |
| | 118– | 250/363 | 0.78 (0.62, 0.98) | 0.93 (0.71, 1.21) |
| | 166+ | 222/395 | 0.63 (0.50, 0.80) | 0.90 (0.69, 1.17) |
| *P* value for trend | | | 0.002 | 0.75 |
| Green vegetables (g day$^{-1}$) | <10 | 10/6 | 2.71 (0.98, 7.53) | 1.75 (0.57, 5.34) |
| | 10– | 89/74 | 2.06 (1.49, 2.86) | 1.59 (1.09, 2.30) |
| | 20– | 599/1016 | 1.00[c] | 1.00[c] |
| | 50+ | 284/390 | 1.23 (1.02, 1.48) | 1.38 (1.11, 1.70) |
| *P* value for trend | | | 0.24 | 0.40 |
| Tomatoes (g day$^{-1}$) | <16 | 310/329 | 1.00 | 1.00 |
| | 16- | 226/370 | 0.66 (0.53, 0.83) | 0.77 (0.59, 0.99) |
| | 27- | 233/403 | 0.62 (0.50, 0.78) | 0.69 (0.54, 0.90) |
| | 45+ | 213/384 | 0.59 (0.47, 0.75) | 0.74 (0.57, 0.96) |
| *P* value for trend | | | <0.001 | 0.01 |
| Carrots (serving) | Never | 65/43 | 1.00 | 1.00 |
| | Monthly or less | 98/102 | 0.62 (0.38, 1.00) | 0.62 (0.36, 1.08) |
| | Weekly | 274/327 | 0.55 (0.36, 0.84) | 0.68 (0.42, 1.10) |
| | More than weekly | 545/1014 | 0.36 (0.24, 0.53) | 0.49 (0.31, 0.78) |
| *P* value for trend | | | <0.001 | <0.001 |
| Butter (teaspoon) | Never | 272/564 | 1.00 | 1.00 |
| | Up to weekly | 118/219 | 1.12 (0.86, 1.47) | 1.14 (0.84, 1.55) |
| | Up to daily | 158/194 | 1.69 (1.31, 2.19) | 1.66 (1.24, 2.23) |
| | More than daily | 434/509 | 1.77 (1.46, 2.15) | 1.44 (1.16, 1.80) |
| *P* value for trend | | | <0.001 | <0.001 |
| Cheese (one slice or one oz) | Monthly or less | 151/221 | 1.00 | 1.00 |
| | Weekly | 199/300 | 0.97 (0.74, 1.28) | 0.93 (0.67, 1.28) |
| | Few times per week | 478/727 | 0.98 (0.77, 1.24) | 0.99 (0.75, 1.30) |
| | Daily or more | 154/238 | 0.95 (0.71, 1.27) | 1.00 (0.71, 1.40) |
| *P* value for trend | | | 0.74 | 0.86 |
| Eggs (one egg) | Monthly or less | 127/195 | 1.00 | 1.00 |
| | Weekly | 185/385 | 0.73 (0.55, 0.97) | 0.85 (0.61, 1.19) |
| | Few times per week | 562/804 | 1.06 (0.83, 1.36) | 1.08 (0.80, 1.44) |
| | Daily or more | 108/102 | 1.62 (1.13, 2.32) | 1.53 (1.02, 2.31) |
| *P* value for trend | | | 0.001 | 0.02 |
| Liver (serving) | Never | 283/489 | 1.00 | 1.00 |
| | Few times per year | 179/290 | 1.06 (0.84, 1.35) | 1.05 (0.80, 1.38) |
| | Monthly | 266/476 | 0.96 (0.78, 1.19) | 0.99 (0.78, 1.26) |
| | Weekly or more | 254/231 | 1.90 (1.51, 2.40) | 1.68 (1.29, 2.19) |
| *P* value for trend | | | <0.001 | 0.002 |
| Margarine (teaspoon) | Never | 231/241 | 1.00 | 1.00 |
| | Up to few times per week | 81/85 | 1.02 (0.71, 1.46) | 0.94 (0.63, 1.42) |
| | Daily | 221/299 | 0.77 (0.60, 1.00) | 0.92 (0.68, 1.23) |
| | More than daily | 449/861 | 0.54 (0.43, 0.67) | 0.67 (0.52, 0.85) |
| *P* value for trend | | | <0.001 | 0.001 |
| Milk (pint) | Weekly or less | 65/102 | 1.00 | 1.00 |
| | Few times per week | 524/909 | 0.90 (0.65, 1.26) | 0.86 (0.59, 1.27) |
| | Daily | 337/443 | 1.22 (0.87, 1.72) | 1.09 (0.73, 1.63) |
| | More than daily | 56/32 | 2.83 (1.65, 4.85) | 2.10 (1.13, 3.89) |
| *P* value for trend | | | <0.001 | 0.003 |

Table continued overleaf

*© 2001 Cancer Research Campaign* 

**Table 2** Continued

| | | | | |
|---|---|---|---|---|
| Tomato sauce (serving) | Never | 559/738 | 1.00 | 1.00 |
| | Few times per year | 64/120 | 0.71 (0.51, 0.98) | 0.79 (0.54, 1.15) |
| | Monthly | 148/253 | 0.78 (0.62, 0.99) | 0.77 (0.59, 1.01) |
| | Few times per week or more | 211/375 | 0.75 (0.62, 0.93) | 0.69 (0.55, 0.87) |
| *P* value for trend | | | 0.003 | 0.001 |
| Vitamin pills (regularly) | No | 913/1321 | 1.00 | 1.00 |
| | Yes | 69/165 | 0.61 (0.45, 0.82) | 0.67 (0.48, 0.94) |
| *P* value for difference | | | 0.001 | 0.02 |
| Fish liver oil (regularly) liquid or capsules | No | 873/1193 | 1.00 | 1.00 |
| | Yes | 109/293 | 0.50 (0.39, 0.63) | 0.60 (0.46, 0.79) |
| *P* value for difference | | | <0.001 | <0.001 |

[a]Adjusted for age in 5-year groups, separately for males and females. [b]Adjusted for age as above, and also for smoking status separately for males and females. [c]Baseline category. [d]95% confidence interval. [e] Total of carotine rich vegetables adjustment for smoking status (see Table 2). For carrots, margarine and tomato sauce respectively, those in the highest intake category had RRs of 0.49 (95% CI 0.31, 0.78), 0.67 (95% CI 0.52, 0.85), and 0.69 (95% CI 0.55, 0.87) compared with those in the lowest category, while subjects who took vitamin pills or fish liver oil had RRs of 0.67 (95% CI 0.47, 0.94) and 0.60 (95% CI 0.46, 0.79) compared with those who did not. There were positive associations for butter, eggs, liver, and milk which remained statistically significant after adjusting for smoking status ($P < 0.001$, $P = 0.02$, $P = 0.002$, and $P = 0.003$ respectively). Those in the highest intake category had RRs of 1.44 (95% CI 1.16, 1.80), 1.53 (95% CI 1.02, 2.31), 1.68 (95% CI 1.29, 2.19) and 2.10 (95% CI 1.13, 3.89) respectively compared with those in the lowest category. For cheese there was no evidence of an association with lung cancer risk. The analysis for milk was repeated considering whole milk and skimmed milk separately and this showed that whole milk was associated with a significant increase in risk (RRs: 1.00, 1.00, 1.24, 2.64; $P = 0.005$), whereas skimmed milk was not (RRs: 1.00, 0.73, 0.83, 0.82; $P = 0.91$).

### Social class

After adjustment for age, sex and smoking, the relative risk of lung cancer among individuals in social class III compared with social classes IV and V was 1.05 (95% CI 0.84, 1.32) while for social classes I and II it was 0.66 (95% CI 0.51, 0.86). These values did not change appreciably when adjustment was made for all 15 dietary variables simultaneously. In order to see whether the dietary differences found above could be explained in terms of social class, the analysis shown in Table 2 was repeated for each dietary variable but including social class as well as age, sex and smoking habits in the model. For most variables the evidence of an association was little changed. However, for carotene, tomatoes and vitamin pills the significance level of the trend became appreciably less extreme, changing from $P = 0.06$, 0.01, and 0.02 to $P = 0.12$, 0.06, and 0.05.

### All dietary variables considered simultaneously

In the analyses described above, each nutrient, food, or supplement was considered separately. When the analysis shown in Table 2 was repeated including all the dietary variables, as well as adjusting for age, sex, smoking, and social class, five dietary variables remained significantly ($P < 0.01$) associated with lung cancer risk (see Table 3). Pre-formed retinol was positively associated with lung cancer risk ($P = 0.0006$) and the RR for subjects in the highest intake quartile was 2.89 (95% CI 1.38, 6.03) compared with subjects in the lowest quartile. Fish liver oil ($P = 0.0001$), vitamin pills ($P = 0.0006$), carrots ($P = 0.0066$) and tomato sauce ($P = 0.0096$) were negatively associated with lung cancer and takers of fish liver oil and vitamin pills had RRs of 0.49 (95% CI 0.33, 0.71) and 0.56 (95% CI 0.36, 0.88) respectively compared with non-takers, while the highest intake categories of carrot and tomato sauce consumption had RRs of 0.60 (95% CI 0.33, 1.08) and 0.74 (95% CI 0.56, 0.98) respectively, compared with subjects in the lowest categories. When this analysis was repeated omitting the 542 subjects who were takers of fish liver oil or vitamin pills one year before the interview, pre-formed retinol remained significantly positively ($P = 0.0003$) and carrots remained significantly negatively ($P = 0.009$) associated with lung cancer risk, while for tomato sauce the significance of the association with lung cancer risk was reduced ($P = 0.034$), and none of the other dietary variables was significantly associated with lung cancer risk at the 1% level. When takers of fish liver oil or vitamin pills were considered on their own, none of the associations with the other dietary variables reached statistical significance at the 1% level.

### Duration of consumption of vitamin pills and fish liver oil

In order to investigate further the decrease in lung cancer risk associated with taking fish liver oil, subjects who had taken it at any time during the 10 years prior to interview were classified into 4 groups according to the length of time that they had taken it. After adjusting for age, sex, smoking, social class and the other dietary variables, subjects who had taken fish liver oil for 1–2, 3–4, 5–9 and 10 years respectively had RRs of 0.58, 0.70, 0.52, and 0.48 compared with subjects who had never taken it in the previous 10 years ($P$ for trend = 0.007, see Table 4). When the analysis was repeated excluding the subjects who had not taken fish liver oil in the previous 10 years, the trend in RR with the number of years that fish liver oil had been taken was no longer significant ($P = 0.42$), although subjects in the two longest duration categories had lower risks than those in the two shortest duration categories (see Table 4). An equivalent analysis for vitamin pills showed no evidence of a trend with increasing duration of consumption (see Table 4).

### DISCUSSION

In agreement with almost all previous observational studies (Ziegler et al, 1996; Department of Health, 1998), we observed

 © 2001 Cancer Research Campaign

**Table 3** Relative risk of lung cancer by categories of food frequency based on usual habits one year before interview. All the nutrients, foods and supplements shown in Table 2 were included simultaneously in the regression and the variables shown below were the only ones for which there was a significant (*P*< 0.01) trend

| Nutrient, food or supplement | Categories[a] | Relative risk adjusted for age, sex, smoking, social class and other dietary variables[b] | *P* value for trend[b] |
|---|---|---|---|
| Pre-formed retinol (µg day$^{-1}$) | <430 | 1.00[c] | 0.0006 |
| | 430– | 1.51 (1.08, 2.12)[d] | |
| | 755– | 1.71 (1.09, 2.69) | |
| | 1536+ | 2.89 (1.38, 6.03) | |
| Fish liver oil | No | 1.00 | 0.0001 |
| | Yes | 0.49 (0.33, 0.71) | |
| Vitamin pills | No | 1.00 | 0.0006 |
| | Yes | 0.56 (0.36, 0.88) | |
| Carrots (serving) | Never | 1.00 | 0.0066 |
| | Monthly or less | 0.62 (0.35, 1.09) | |
| | Weekly | 0.77 (0.47, 1.28) | |
| | More than weekly | 0.60 (0.33, 1.08) | |
| Tomato sauce (serving) | Never | 1.00 | 0.0096 |
| | Few times per year | 0.81 (0.54, 1.20) | |
| | Monthly | 0.83 (0.62, 1.11) | |
| | Few times per week or more | 0.74 (0.56, 0.98) | |

[a]Numbers of cases and controls in each category as in Table 2. [b]Adjusted for age, sex and smoking as in right hand column of Table 2 and also for social class. Each dietary variable also adjusted for all other dietary variables shown in Table 2. [c]Baseline category. [d]95% confidence interval.

**Table 4** Lung cancer odds ratios by duration of consumption of vitamin pills and fish liver oil in previous 10 years

| | Duration of consumption in previous 10 years (years) | No. of cases/ No. of controls | Relative risk adjusted for age, sex, smoking, social class and all other dietary variables listed in Table 2 | |
|---|---|---|---|---|
| | | | Including those with 0 years consumption | Excluding those with 0 years consumption |
| Fish oil | 0 | 852/1161 | 1.00[a] | – |
| | 1–2 | 73/174 | 0.58 (0.39, 0.87)[b] | 1.00[a] |
| | 3–4 | 27/54 | 0.70 (0.38, 1.29) | 1.19 (0.57, 2.48) |
| | 5–9 | 17/56 | 0.52 (0.27, 1.00) | 0.64 (0.28, 1.46) |
| | 10 | 13/41 | 0.48 (0.22, 1.05) | 0.84 (0.34, 2.10) |
| *P* value for trend | | | <0.007 | 0.42 |
| Vitamin pills | 0 | 904/1304 | 1.00[a] | – |
| | 1–2 | 37/71 | 0.62 (0.36, 1.06) | 1.00[a] |
| | 3–4 | 13/41 | 0.41 (0.19, 0.86) | 0.64 (0.14, 2.84) |
| | 5–9 | 18/34 | 0.76 (0.36, 1.57) | 2.24 (0.47, 10.67) |
| | 10 | 10/36 | 0.67 (0.28, 1.59) | 0.44 (0.08, 2.41) |
| *P* value for trend | | | 0.08 | 0.75 |

[a]Baseline category. [b]95% confidence interval.

moderate but highly statistically significant negative associations between several carotenoid rich foods and lung cancer risk, which were reduced but not eliminated by adjustment for smoking. We also observed an increased risk of lung cancer associated with retinol rich foods as found in some, but not all, previous studies (Ziegler et al, 1996; Department of Health, 1998; Willett, 1998). The principal issue is whether the results imply that certain foods reduce or increase the risk of lung cancer, or are they artefact?

The information provided by this study is determined by its size, the accuracy of the dietary assessment, the participation rates among cases and controls, their representativeness of the underlying population, and the effectiveness of the statistical adjustment for smoking. The study was large, giving high power to detect moderate associations of dietary factors with lung cancer risk and the associations reported cannot all be attributed to chance. The questionnaire on vitamin A intake was not itself validated but was similar to a validated questionnaire used previously in a similar English population (Harris et al, 1991). Most other food frequency questionnaires designed to assess carotene intake have shown similar moderate correlations with serum carotenoids (Willett and Lenart, 1998), and it is likely that the errors in assessing intake of carotene and related foods are largely random and will therefore cause underestimation of true associations, rather than introduce spurious associations.

In this study, cases were individuals with newly diagnosed lung cancer identified by surveillance of all the hospitals responsible for the treatment of lung cancer in a geographically isolated area and, after matching for age, sex and broad area of residence, controls were selected at random from population lists that were essentially complete. Thus cases and controls were both drawn from the same

© 2001 Cancer Research Campaign 

underlying population. The proportion of eligible cases who were not eventually included in the study was 12% and the most common reason for exclusion was because they were too ill or had died (Darby et al, 1998), which is unlikely to be strongly related to their previous diet. The proportion of eligible controls who were not eventually included in the study was 21% and the most common reason for exclusion was subject refusal. It is therefore possible that those who agreed to be interviewed had diets that differed from those who did not. Koo (1997) has suggested that the dietary factors associated with risk in most studies may be markers of a 'health conscious' dietary pattern within the population studied. This has varied somewhat with place and time. For example, in two studies at the Roswell Park Cancer Institute, the first in 1957–1965 and the second in 1982–1987, the first observed that high milk consumption, which would have been perceived at that time to be 'healthy', was associated with a reduction in risk, whereas the second observed that whole milk intake was associated with an increase in risk whereas low fat milk was associated with a reduction in risk (reviewed by Koo, 1997; it should be noted that we also observed an increase in risk associated with increasing consumption of whole milk but not of low fat milk – see above). It is possible that high consumption of carrots and tomato sauce and use of fish liver oil supplements and vitamin pills, and also low consumption of retinol-rich foods (such as liver, butter, eggs and whole milk), as observed in the controls, may be markers of a 'health conscious' lifestyle among elderly men and women in south-west England around 1990, and that individuals with such a lifestyle were more likely to agree to take part in the study as controls than others. We cannot examine this directly, but in 1990 health conscious individuals would have had avoidance of cigarette-smoking as a priority. Some evidence as to the likely magnitude of any such effect is therefore available by comparing the smoking status of the population controls in this study with that of the hospital controls, who comprised individuals admitted for diseases thought not to be related to smoking, and where the proportion of eligible individuals who were not included in the study was only 4% (Darby et al, 1998). Smoking patterns in the 2 groups were very similar, with only minor differences that were too small to have a material impact on the results.

It is also possible that unhealthy patients are biased in their reporting of quantitative food habits compared with healthy controls (Friedenreich et al, 1991; Willett, 1998). However, it does not seem plausible that such a bias could be sufficient to produce the various differences recorded, although in the absence of objective measurements such biases cannot be wholly excluded.

It is clear that the commonly observed association between carotenoid rich foods and lung cancer risk is partially confounded by smoking, since it is well established that smokers have a lower intake of carotene rich foods than non-smokers (Margetts and Jackson, 1993). In the current study, as in most others, adjustment for smoking reduced but did not eliminate the association between lung cancer risk and certain carotene rich foods. Given the imperfect measurement of smoking history in most epidemiological studies, it remains possible that the reported associations with diet could often be partly due to residual confounding by smoking. However, the information collected on smoking in this study was exceptionally detailed, and adjustment for smoking in 16 categories rather than 7 produced no further attenuation of the RRs observed in relation to carotenoid rich foods. There were only 26 subjects with lung cancer in our study who had never smoked, which was too few to provide useful data on diet and lung cancer among never smokers.

Several previous case–control investigations have studied substantial numbers of never smokers and have observed reductions in lung cancer risk in association with high intakes of fruits and vegetables (Brownson et al, 1998; Brennan et al, 2000). The similar results obtained by these studies of never smokers and studies which included smokers but adjusted for the effect of smoking also suggests that residual confounding by smoking is unlikely to explain the associations. The results of prospective studies have generally supported the conclusion that high intakes of fruits and vegetables are associated with a reduction in the risk for lung cancer, although the results for vegetables are less consistent than those from case–control studies (Department of Health, 1998). 2 prospective studies which reported on *never* smokers have only small numbers of lung cancers and are inconclusive (Fraser et al, 1991; Voorrips et al, 2000).

In our study, even after adjusting for smoking, for social class, and for all the other dietary factors simultaneously, we still observed statistically significant and apparently independent associations of lung cancer risk with pre-formed retinol (increase in risk with increased intake), and with fish liver oil, vitamin pills, carrots, and tomato sauce (decreases in risk with increase in intake). These specific associations have all been observed in at least some previous studies. For example, the recent reports of Nyberg et al (1998) and Speizer et al (1999) described highly significant reductions in risk associated with increasing carrot consumption, perhaps because this food is a good marker for a diet rich in vegetables. Giovannucci (1999) showed that the majority of studies have observed some reduction in risk with increasing tomato consumption, and argued that this might be due to a protective effect of lycopene; it is therefore interesting to note that in our study tomato sauce appeared to be more strongly related to risk than fresh tomatoes, and laboratory studies have shown that the lycopene in tomato sauce is much more easily absorbed than the lycopene in raw tomatoes. A possible protective effect of cod liver oil was previously observed in a prospective study in Norway and it was suggested that this could be due to n-3 fatty acids in fish liver oil (Veierød et al, 1997). However, the lack of any association in our study between duration of use and lung cancer risk among takers of either fish liver oil or vitamin pills, suggests that these associations are due to confounding rather than causal. Speizer et al (1999) observed reductions in risk associated with use of vitamin supplements but, as in the present study, there was no clear association with increasing duration of use.

The strongest dietary association in the present study was the increase in risk with increasing consumption of retinol. Mayne et al (1991) suggested that dietary retinol might promote carcinogenesis at several sites, while β-carotene and retinol given together in the CARET trial was associated with a 28% (95% CI 4, 57) increase in lung cancer risk (Omenn et al, 1996). Prospective studies of serum retinol and lung cancer, however, have not suggested that high levels of retinol increase risk (Wald et al, 1980; Willett, 1998). In the present study, the inverse associations of risk with two rich supplementary sources of retinol (vitamin pills and fish liver oil), as well as the positive association with retinol, suggest that at least some of these associations may be due to confounding, rather than being causal. It is possible that adjustment for fat consumption might remove the retinol association in this population, but such adjustment was not possible given the data collected.

Overall, our results are similar to those of previous observational studies. We now know from the results of the randomized

© 2001 Cancer Research Campaign trials that β-carotene itself is unlikely to protect against lung cancer. Whilst some of the associations that we have observed may be causal, it is unlikely that those observed with retinol, fish liver oil, vitamin pills, carrots and tomato sauce are all due to *independent* biological effects on cancer development because this would require that 5 different nutritional factors each have an independent effect each with a similar order of magnitude of effect. We therefore conclude that there is at least one as yet unidentified factor that is causally related to lung cancer risk and of considerable importance in terms of attributable risk (see Table 3). Any such factor, or factors, are probably dietary, although not necessarily so, and would be negatively associated with intake of fish liver oil, vitamin pills, carrots or tomato sauce, or positively associated with intake of retinol in this population.

### ACKNOWLEDGEMENTS

We thank all the individuals and research assistants who took part in this study, the staff in hospitals and general practices for assistance, and Anthea Craven for secretarial assistance. The study was carried out in the ICRF Cancer Epidemiology Unit and was funded by the ICRF, the National Radiological Protection Board, the Department of Health, the Department of the Environment Transport and the Regions, and the European Commission.

### REFERENCES

Alpha-Tocopherol, Beta Carotene Cancer Prevention Study Group (1994) The effect of vitamin E and beta carotene on the incidence of lung cancer and other cancers in male smokers. *N Engl J Med* **330**: 1029–1035

Bjelke E (1975) Dietary vitamin A and human lung cancer. *Int J Cancer* **15**: 561–565

Brennan P, Fortes C, Butler J, Agudo A, Benhamou S, Darby S, Gerken M, Jöckel K-H, Kreuzer M, Mallone S, Nyberg F, Pohlabeln H, Ferro G and Boffetta P (2000) A multicenter case-control study of diet and lung cancer among non-smokers. *Cancer Causes Control* **11**: 49–58

Brownson RC, Alavanja MCR, Caporaso N, Simoes EJ and Chang JC (1998) Epidemiology and prevention of lung cancer in nonsmokers. *Epidemiol Rev* **20**: 218–236

Cook NR, Lee IM, Manson JAE, Buring JE and Hennekens CH (2000) Effects of beta-carotene supplementation on cancer incidence by baseline characteristice in the Physicians' Health Study (United States). *Cancer Causes Control* **11**: 617–626

Darby S, Whitley E, Silcocks P, Thakrar B, Green M, Lomas P, Miles J, Reeves G, Fearn T and Doll R (1998) Risk of lung cancer associated with residential radon exposure in south-west England: a case-control study. *Br J Cancer* **78**: 394–408

Department of Health (1998) Nutritional Aspects of the Development of Cancer. In: *Report on Health and Social Subjects*, **48**: 95–108. The Stationery Office

Fraser GE, Beeson WL and Phillips RL (1991) Diet and lung cancer in California Seventh-Day Adventists. *Am J Epidemiol* **133**: 683–693

Friedenreich CM, Howe GR and Miller AB (1991) An investigation of recall bias in the reporting of past food intake among breast cancer cases and controls. *Ann Epidemiol* **1**: 439–453

Giovannucci E (1999) Tomatoes, tomato-based products, lycopene, and cancer: review of the epidemiologic literature. *J Natl Cancer Inst* **91**: 317–331

Harris RWC, Key TJA, Silcocks PB, Bull D and Wald NJ (1991) A case-control study of dietary carotene in men with lung cancer and in men with other epithelial cancers. *Nutr Cancer* **15**: 63–68

Hennekens CH, Buring JE, Manson J-AE, Stampfer M, Rosner B, Cook NR, Belanger C, LaMotte F, Gaziano JM, Ridker PM, Willett W and Peto R (1996) Lack of effect of long-term supplementation with beta carotene on the incidence of malignant neoplasms and cardiovascular disease. *N Engl J Med* **334**: 1145–1149

Holland B, Welch AA, Unwin ID, Buss DH, Paul AA and Southgate DAT (1991) *McCance and Widdowson's The Composition of Foods*, 5th edn. Royal Society of Chemistry: Cambridge

Koo LC (1997) Diet and lung cancer 20+ years later: more questions than answers? *Int J Cancer* **Suppl 10**: 22–29

Lee I-M, Cook NR, Manson J-AE, Buring JE and Hennekens CH (1999) β-carotene supplementation and incidence of cancer and cardiovascular disease: the Women's Health Study. *J Natl Cancer Inst* **91**: 2102–2106

Margetts BM and Jackson AA (1993) Interactions between people's diet and their smoking habits: the dietary and nutritional survey of British adults. *Br Med J* **307**: 1381–1384

Mayne ST, Graham S and Zheng T (1991) Dietary retinol: prevention or promotion of carcinogenesis in humans? *Cancer Causes Control* **2**: 443–450

Ministry of Agriculture, Fisheries and Food (1993) *Food portion sizes*, 2nd edn. HMSO: London

Nyberg F, Agrenius V, Svartengren K, Svensson C and Pershagen G (1998) Dietary factors and risk of lung cancer in never-smokers. *Int J Cancer* **78**: 430–436

Office of Population Censuses and Surveys (1980) *Classification of Occupations*. HMSO: London

Omenn GS, Goodman GE, Thornquist MD, Balmes J, Cullen MR, Glass A, Keogh JP, Meyskens FL, Valanis B, Williams JH, Barnhart S and Hammar S (1996) Effects of a combination of beta carotene and vitamin A on lung cancer and cardiovascular disease. *N Eng J Med* **334**: 1150–1155

Peto R, Doll R, Buckley JD and Sporn MB (1981) Can dietary beta-carotene materially reduce human cancer rates? *Nature* **290**: 201–208

Peto R, Darby S, Deo H, Silcocks P, Whitley E and Doll R (2000) Smoking, smoking cessation, and lung cancer in the UK since 1950: combination of national statistics with two case-control studies. *Br Med J* **321**: 323–329

Proprietary Association of Great Britain (1996) *OTC Directory 96/97*. Communications International Group

Speizer FE, Colditz GA, Hunter DJ, Rosner B and Hennekens C (1999) Prospective study of smoking, antioxidant intake, and lung cancer in middle-aged women (USA). *Cancer Causes Control* **10**: 475–482

StataCorp (1997) *Stata Statistical Software: Release 5.0*. Stata Corporation: College Station, TX

Veierød MB, Laake P and Thelle DS (1997) Dietary fat intake and risk of lung cancer: a prospective study of 51,452 Norwegian men and women. *Eur J Cancer Prev* **6**: 540–549

Voorrips LE, Goldbohm RA, Verhoeven DTH, van Poppel GAFC, Sturmans F, Hermus RJJ and van den Brandt PA (2000) Vegetable and fruit consumption and lung cancer risk in the Netherlands Cohort Study on Diet and Cancer. *Cancer Causes Control* **11**: 101–115

Wald NJ, Idle M, Boreham J and Bailey A (1980) Low serum-vitamin-A and subsequent risk of cancer. *Lancet* **2** 813–815

Willett W (1998) *Nutritional Epidemiology*, 2nd Edition, pp. 364, 396. New York: Oxford University Press

Willett W and Lenart E (1998) Reproducibility and validity of food-frequency questionnaires. In: Willett W (ed.) *Nutritional Epidemiology*, 2nd edn. pp 101–147. Oxford University Press: New York

World Health Organization (1975) ICD-9. *International Classification of Diseases*. WHO: Geneva

Ziegler RG, Mayne ST and Swanson CA (1996) Nutrition and lung cancer. *Cancer Causes Control* **7**: 157–177

*© 2001 Cancer Research Campaign*
